# MicroRNA Controlled Adenovirus Mediates Anti-Cancer Efficacy without Affecting Endogenous MicroRNA Activity

**DOI:** 10.1371/journal.pone.0016152

**Published:** 2011-01-10

**Authors:** Ryan Cawood, Suet-Ling Wong, Ying Di, Dilair F. Baban, Leonard W. Seymour

**Affiliations:** 1 Medicinal Virology Research Group, Department of Clinical Pharmacology, University of Oxford, Oxford, United Kingdom; 2 Genomics Group, Wellcome Trust Centre for Human Genetics, University of Oxford, Oxford, United Kingdom; IBMC-CNRS, France

## Abstract

MicroRNAs are small non-coding RNA molecules that regulate mRNA translation and stability by binding to complementary sequences usually within the 3′ un-translated region (UTR). We have previously shown that the hepatic toxicity caused by wild-type Adenovirus 5 (Ad5WT) in mice can be prevented by incorporating 4 binding sites for the liver-specific microRNA, mir122, into the 3′ UTR of E1A mRNA. This virus, termed Ad5mir122, is a promising virotherapy candidate and causes no obvious liver pathology. Herein we show that Ad5mir122 maintains wild-type lytic activity in cancer cells not expressing mir122 and assess any effects of possible mir122 depletion in host cells. Repeat administration of 2×10^10^ viral particles of Admir122 to HepG2 tumour bearing mice showed significant anti-cancer efficacy. RT-QPCR showed that E1A mRNA was down-regulated 29-fold in liver when compared to Ad5WT. Western blot for E1A confirmed that all protein variants were knocked down. RT-QPCR for mature mir122 in infected livers showed that quantity of mir122 remained unaffected. Genome wide mRNA microarray profiling of infected livers showed that although the transcript level of >3900 different mRNAs changed more than 2-fold following Ad5WT infection, less than 600 were changed by Ad5mir122. These were then filtered to select mRNAs that were only altered by Ad5mir122 and the remaining 21 mRNAs were compared to predicted mir122 targets. No mir122 target mRNAs were affected by Ad5 mir122. These results demonstrate that the exploitation of microRNA regulation to control virus replication does not necessarily affect the level of the microRNA or the endogenous mRNA targets.

## Introduction

Genetic regulation of virus replication and expression is a powerful strategy for the development of safer viral therapeutics for vaccination, gene therapy and virotherapy. DNA viruses are often made tissue or cancer selective by the replacement of strong viral promoters with weaker human endogenous promoters. In contrast, mechanisms for the control of RNA virus replication have largely focussed on engineering or exploiting interferon sensitivity [1], [2] or using attenuated vaccine strains [3]. No universal mechanism of control for all viruses or viral transgenes has proven successful. However, the discovery of the microRNA regulatory network has allowed the design of viral vectors in which essential mRNAs are selectively degraded in tissues expressing a specific microRNA, affording the possibility of control through tissue-selective inactivation of essential viral functions.

MicroRNAs are non-coding RNA molecules that negatively regulate mRNA translation through binding to sequences usually found within the 3′ un-translated region (UTR) [4]. The level of complementarity between the microRNA and the target influences whether the mRNA is degraded (perfect complementarity) or forms a stable translationally inactive complex. The inclusion of microRNA binding sites into the 3′UTR of mRNAs encoding essential viral proteins allows the selective destruction of the mRNA and prevents protein translation. The pre-requisite of all viruses to exploit mRNA translation in order to replicate means that this method of control can be universally applied to every virus infecting a eukaryotic cell.

Examples in which the technique has been used successfully are in the control the of replication of Polio virus [5], Herpesvirus [6], Vesicular Stomatitis virus [7], [8], Influenza virus [9] and Adenovirus type 5 (Ad5) [10], [11]. Viral vectors in which this technique has been successfully used to control transgene expression include Dengue virus replicons [12], Alphaviral replicons [13], Lentiviral vectors [14] and Adenoviral vectors [15]. Based on current literature, it is clear that the virus type, the microRNA of choice and the tissue of pathology are all important in defining the level to which a virus is successfully controlled.

Ad5 has been extensively investigated in the context of virotherapy and the wild type strain (Ad5WT) has been shown to have potent anticancer activity [16]. However, following intravenous administration to mice, Ad5WT causes acute hepatic toxicity which, at doses above 1x10^9^ pfu, is uniformly fatal [17]. This toxicity is believed to reflect high levels of expression of the E1A protein in hepatocytes [18], [19]. This expression is then followed by downstream viral gene expression and hepatocyte cell death.

To abrogate this toxicity, without affecting viral replication in cancer cells, we inserted four perfectly complementary binding sites for the hepatocyte-specific microRNA mir122 into the 3′ UTR of the E1A transcription cassette (a virus termed Ad5mir122). The perfect complementarity between the mir122 binding sites we have inserted and microRNA mir122 should result in substantial E1A mRNA cleavage through a mechanism similar to RNAi. We have shown previously that this can reduce hepatic viral replication, ALT release and liver pathology [10].

Little is known about the potential unwanted cellular consequences of the control of viruses using microRNA binding sites. The amount of cellular microRNA could be decreased by the addition of viral target mRNAs or the viral mRNAs could saturate the activity of the microRNA and allow changes in the levels of endogenous mRNA targets. Previous studies using microRNA regulated lentiviral vectors have shown that this can occur when cells are exposed to a high multiplicity of infection. This suggests that the regulatory effect of microRNAs are saturable at high doses [20]. Below this threshold the microRNA endogenous mRNA targets are reported to remain unchanged [21]. In this study we set out to determine whether the dose of Ad5mir122 capable of showing intravenous efficacy in treating cancer would significantly affect hepatic levels of mir122 or its mRNA targets.

## Materials and Methods

### Adenovirus preparations

Adenovirus cloning has been described previously [10]. All adenoviruses were grown in A549 cells, purified by double banding in CsCl gradients with benzonase treatment after the first banding. Viral particle (vp) number was determined by measuring DNA content using a modified version of the PicoGreen assay (Invitrogen, Paisley, UK) [22]. TCID_50_ calculated with the KÄRBER statistical method [23] was used to estimate the adenovirus titer (TCID_50_ units/ml) and corrected to determine plaque forming units/ml (pfu/ml). Adenovirus preparation characteristics are as follows: Ad5WT: 1.13×10^12^ vp/ml, 1.98×10^11^ pfu/ml and particle:infectivity (P:I) ratio of 5.6; Ad5mir122: 1.29×10^12^ vp/ml, 2.01×10^11^ pfu/ml and particle:infectivity (P:I) ratio of 6.4.

### Maintenance of cell lines

HT29-Luc and Lovo-Luc colorectal cell lines were obtained from Caliper Life Sciences (Runcorn, UK). Human hepatocellular carcinoma HepG2 and Hep3b cell lines and A549 lung carcinoma cells were obtained from the European Collection of Cell Cultures (Porton Down, UK), and maintained in DMEM with 10% foetal bovine serum (FBS) (PAA Laboratories, Yeovil, UK) including penicillin (25 U/ml) and streptomycin (10 mg/ml).

### Luciferase assays

Cells were seeded in triplicate in 48 well plates at 2.5×10^4^ cells/well. Virus infections were performed at 10 vp/cell. 24 hrs following infection the media was removed and 150 µl reporter cell lysis buffer (Promega) was added. Cells were frozen at −80°C for 1 hr before thawing. Luciferin (25 µl) (Promega, Southampton, UK) was added to 25 µl of cell lysate and relative luminescence was measured by luminometry (Lumat LB 9507, Berthold Technologies, Redbourn, UK).

### MTS Assays

Cell viability was determined using the CellTiter 96 Aqueuos One Solution Cell Proliferation Assay (Promega). Cells were seeded at 1×10^4^/well in 96 well plates in 100 µl media. After 24 hours, viruses were added at 10 and 100 vp/cell in 100 µl media. Cells were left for either 3 or 6 days. At each time point media was removed and 20 µl MTS reagent in 180 µl media was added and cells were incubated for 30 minutes. Plates were read at 490 nm for 0.1 seconds. Blank wells containing MTS reagent and media but no cells served as background and non-infected cells represent 100% survival in each cell type. N = 5 for each assay.

### Primary Hepatocyte culture

Frozen Human primary hepatocytes (NHeps, CC-2591), Hepatocyte Basal medium HBM^TM^(CC-3199), supplements and growth factors HCM^TM^ SingleQuots (CC-4182) were purchased from Lonza. Cells were seeded and manipulated according to Lonza's protocol. A 96 well plate was coated with rat tail collagen type 1 (Sigma, C3867) at 60 µg/cm^2^. Cells were thawed at 37°C in a water bath, centrifuged at 4°C for 3 minutes at 50 g and then washed in cold culture medium. Cells were re-suspended and the number and viability was confirmed using the Trypan blue dye exclusion method. The viability obtained was 60% at seeding. Cells were seeded at 5×10^4^/well with 120 µl medium each well. The medium was changed at 3 hours after seeding. Virus infections were performed 24 hrs after seeding in 100 µl fresh medium at 1 vp/cell, another 100 µl medium was added at 2 hrs post-infection. 100 µl of media was exchanged each day and until experiment completion (72 hrs). The supernatant was removed by aspiration and cells were lysed in 100 µl Promega reporter lysis buffer (E397A) and frozen at −80°C before luciferase quantitation.

### Western Blots

Protein was extracted from murine liver by homogenisation in Promega lysis solution at 80 mg/ml wet weight. Samples were span at 2000 rpm for 5 minutes at 4°C to remove cell debris. 30 µg of liver protein was loaded onto a 10% polyacrylamide gel after quantitation using a QuantiPro BCA assay (Sigma Aldrich, cat: QPBCA-1KT). The gel was run at 160V for 1 hr and then protein was blotted to a nitrocellulose membrane overnight at 4°C at 30V. Nitrocellulose membrane was stained with Ponceau solution to confirm equal loading (data not shown) and then blocked using 5% milk powder (Fluka, Sigma Aldrich) for 2 hrs (E1A) or overnight (Aldolase A). Membrane was washed twice with PBS 0.1% Tween 20 and then once with PBS. For the E1A western blot, primary antibody recognising E1A (AbCam, Cambridge, UK, cat number: Ab33183) was added at 1∶500 dilution in 2.5% milk powder in PBS for 1 hr. Membrane was washed as above. Secondary anti-rabbit HRP labelled antibody was added at 1∶1000 dilution in 2.5% milk powder for 1 hr. For the Aldolase A western blot, primary antibody recognising Aldolase A (Sigma-Aldrich, cat number: AV48130) was added at 1∶1000 dilution in 2.5% milk powder in PBS for 1 hour. Membrane was washed as above. Secondary antibody was the same as used for Adenovirus E1A western blot but used at a 1∶4000 dilution for 1 hour. The membranes were washed as above and then bathed in ECL western blotting detection reagent (Amersham, GE Healthcare) at 0.125 ml/cm^2^ for 1 minute. Blot was visualised in an Alpha Innotech gel documentation system for 1, 5 and 10 minutes using chemi-luminescence detection.

### RNA extraction and Reverse-transcription quantitative PCR for E1A mRNA

Total RNA from murine livers was extracted using mirVana miRNA isolation kit (Ambion) without the miRNA enrichment step. Reactions were performed using TaqMan RNA-to-C_T_ 1-step kit (Applied Biosystems), following the manufacturers protocol. PCR cycles were as follows: 95°C 10 minutes, then 40 cycles at 95°C 15 seconds, 60°C 1 minute. Primer sequences for targeting E1A 13S were: Fwd – ATG TTT GTC TAC AGT CCT GTG TCT GAA, Rev – GAT AGC AGG CGC CAT TTT AGG and probe – CCA GAA CCG GAG CCT GCA AGA CCT AC (Sigma-Aldrich), dual labeled at the 5′ end with 6-carboxyfluorescein and the 3′ end with 6-carboxytetramethylrhodamine. Results were analysed with StepOne Plus Real-Time PCR Systems software (Applied Biosystems). Standard curves were made by serial dilutions of known quantities of E1A DNA using an Ad5 E1A encoding plasmid and assumes 100% extraction efficiency.

### Animal Models

Nu/nu CD1 female mice were obtained from Charles Rivers Laboratories at 4–6 weeks old. 5×10^6^ HepG2 cells were injected subcutaneously. Mice were randomised prior to treatment. Initial tumour sizes were typically 10–20 mm^3^. All were pre-treated with bisphosphonate liposomes (100 ìl/mouse, obtained from Dr Nico van Rouijen) 24 h before the first dose of virus. In the study of intravenous efficacy mice received bisphosphonate liposomes twice at days -1 and 18. All control animals received this treatment. 10 Animals were used in each group.

### Tumour sizing and Kaplan Meier analysis

Tumour volume was measured using hand-held callipers and is defined as the size of the largest tumour in each mouse. Tumour volume is calculated as an ellipsoid [24]. Kaplan Meier analysis was performed using Prism software and is shown as the percentage group survival at each time point.

### Imaging

*In vivo* virus activity was assessed by non-invasive luciferase imaging using an IVIS 100 system (Xenogen, MA). D-Luciferin (potassium salt) (Gold Biotechnology inc) was prepared in PBS at 15.8 mg/ml. 100 µl Luciferin was administered via intra-peritoneal injection and mice were imaged 4 minutes later.

### Measurement of Serum Alanine Aminotransferase (ALT)

50 µl blood was taken from mice by tail bleed and allowed to clot (15 min, room temperature) and spun at 1200 g for 10 min. Serum was isolated and immediately frozen at −20°C. Samples of thawed serum (5 µl) were added to ALT reagent (995 µl, Microgenics) in a 1 ml quartz cuvette, incubated at 37°C and the change in absorbance (340 nm) per minute was measured. Units of ALT per litre were calculated according to the manufacturer's instructions using the following equation: Activity in Units/Litre  = Ä Absorbance change per minute x factor (factor  =  total reaction volume × 1000/6.3 × sample volume × path length).

### MicroRNA Quantitation

RNA was extracted from murine liver as described above and was diluted to 1 ng/µl in nuclease free water. Mature mir122 levels were quantified using a TaqMan MicroRNA Assay (Applied Biosystems) specific for mir122 (Part number: 4427975 Assay ID: 002130). Briefly, Reverse transcription (RT) reactions (15 µl) were set up according to manufacturer's guidelines to using Multiscribe Reverse Transcriptase (50U/reaction), dNTPs (1 mM final concentration), 0.188 µl RNase inhibitor, 1.5 µl 10X RT buffer, 3 µl 5X mir122 TaqMan MicroRNA RT primer and 5 µl RNA sample (1 ng/µl). Reaction conditions were 30 minutes 16°C, 30 minutes 42°C, 5 minutes 4°C. cDNA (1.33 µl of RT reaction) was added to 1 µl 20X Mir122 Taqman MicroRNA Assay, 10 µl TaqMan 2X Universal PCR master mix and made up to 20 µl using nuclease free water. Real-time PCR conditions were 10 minutes at 95°C, followed by 40 cycles of 15 seconds at 95°C then 1 minute at 60°C. Samples were run on a Step One Plus real-time PCR system (Applied Biosystems). The microRNA let7a was used as an internal standard using the conditions described above and using the TaqMan MicroRNA Assay (Applied Biosystems) specific for mature let7a RNA (Part number: 4427975 Assay ID: 000377). Relative standard curves were produced by ten-fold serial dilutions of liver RNA extracted from a mouse receiving PBS. Reaction properties were as follows; mir122 assay: slope 3.28, 101.78%, efficiency 2.02 and Let 7a assay slope 3.268, 102.29%, efficiency 2.01. Relative expressions were calculated using the method published by Pfaffl, M [25]. Probes were labeled at the 5′ end with 6-carboxyfluorescein and at the 3′ end with a non-fluorescent quencher coupled to a minor groove binder (MGB).

### mRNA microarray analysis

RNA was extracted from murine liver as described above. Each group contains 3 mice and independent chips were used for each sample. Whole-genome gene expression analysis were performed using Illumina Single Colour Mouse WG-6_V2_0_R0_11278593_A BeadChip with direct hybridization assay (Illumina, Essex, UK) according to the manufacturer's directions starting with 300 ng total RNA from each sample. Biotinylated cRNA was prepared using the Illumina TotalPrep-96 RNA Amplification Kit (# 4393543 Ambion, Inc., Austin, TX). This briefly includes a first- and second-strand reverse transcription step, followed by a single *in vitro* transcription (IVT) amplification that incorporates biotin-labelled nucleotides. Subsequent steps include array hybridization, washing, blocking, and streptavadin-Cy3 staining. Fluorescence emissions by Cy3 were quantitatively detected by BeadArray Scanner for downstream analysis. GenomeStudio Data Analysis Software was used to Visualize and analyze data generated. This software provides results in standard file formats that can be readily processed with most commercial expression-analysis software programs. Data was imported to GeneSpring GX 11.0.2 (Agilent Technologies, Inc, Santa Clara CA) normalized with Shift to 75 percentile and baseline transformed to median of all samples for detailed analysis to identify significantly differentially expressed genes. The data discussed in this publication is MIAME compliant and have been deposited in NCBI's Gene Expression Omnibus [26] and are accessible through GEO Series accession number GSE23854 (http://www.ncbi.nlm.nih.gov/geo/query/acc.cgi?acc=GSE23854).

### Ethics Statement

All animal experimentation was performed in accordance with the terms of UK Home Office guidelines and the UKCCCR Guidelines for the Welfare of Animals in Experimental Neoplasia. The home office project license number under which these experiments were conducted is PPL 30/2333. All experiments were conducted with approval of the Medical Sciences Animal Ethics Committee, University of Oxford.

## Results

### Ad5mir122 shows decreased E1A expression in primary human hepatocytes

**Figure 1:**
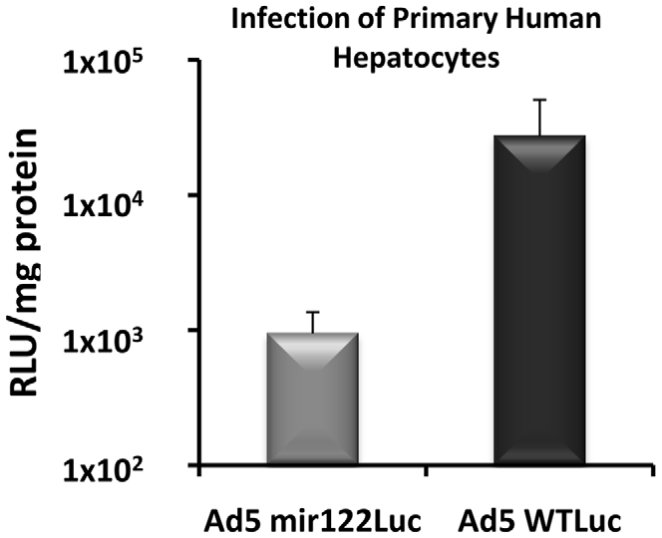
Ad5mir122 shows reduced E1A activity in primary hepatocytes. Primary human hepatocytes were infected with either Ad5mir122luc or Ad5WTluc at 1 vp/cell. Luciferase levels are shown 3 days post infection (n = 3) as relative light units per mg total protein. Error bars represent standard deviation.

We have previously shown that the inclusion of microRNA binding sites within the 3′UTR of adenovirus E1A mRNA leads to lower E1A protein in both murine liver *in vivo* and in the mir122 positive human hepatoma cell line Huh7 *in vitro*. However, Huh7 cells express only 8% of the mir122 levels of primary human hepatocytes [27]. We therefore wanted to measure the level of control of viral transgene expression that could be achieved in primary human hepatocytes as a surrogate for human liver. Primary human hepatocytes (1×10^4^/well) were incubated with either Ad5WT encoding luciferase C-terminally fused to the E1A protein (Ad5WTLuc) or the equivalent virus containing four mir122 binding sites in the E1A-luciferase 3′UTR (Ad5mir122luc). After 72 hours the luciferase levels were >30-fold lower in cells infected with Ad5mir122luc (9.5×10^2^ RLU/mg) when compared to Ad5WTluc (2.7×10^4^ RLU/mg) (Figure 1). This is the first demonstration of control of a microRNA regulated virus in primary human cells and highlights the potential clinical utility of exploiting microRNA regulation in therapeutic viruses.

### Ad5mir122 kills mir122 negative cells with Ad5WT potency

**Figure 2:**
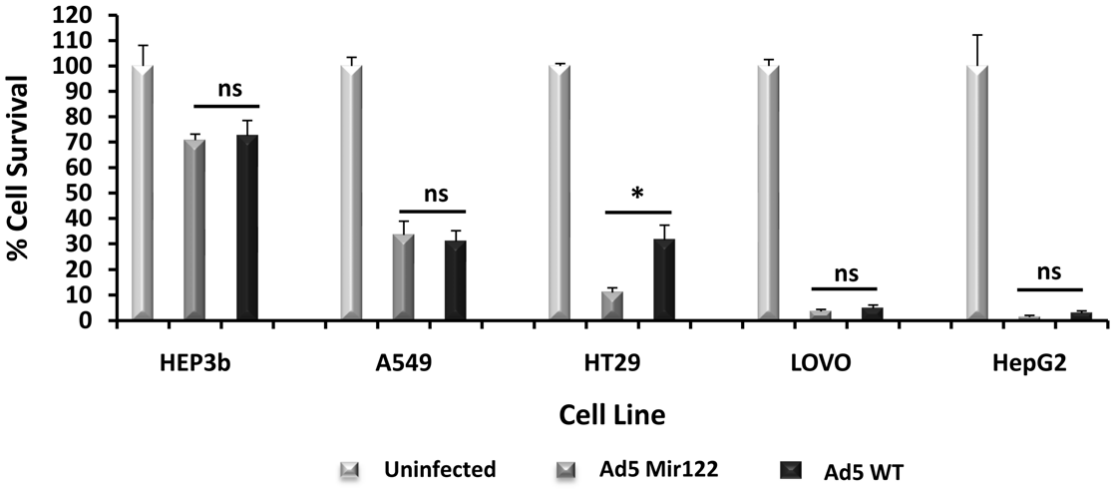
Ad5mir122 kills mir122 negative cells with Ad5WT potency. Comparison of Ad5mir122 and Ad5WT in cancer cell lines incubated at a multiplicity of infection of 100 viral particles per cell. The percentage cell survival is shown 6 days post infection (N = 5) using an MTS cell survival assay. Statistical analysis was performed using one way ANOVA (* = p<0.05, NS  =  not significant).

The ability of Ad5mir122 to kill tumour cells not expressing mir122 was compared to Ad5WT. Multiple cell lines were incubated at 10 and 100 vp/cell and the cell viability was determined after 3 or 6 days by MTS assay. Equal cell killing was observed with both viruses at both MOIs by day 3 (data not shown). Almost complete cell killing in some cell lines was observed at the higher MOI by day 6 (Figure 2). Interestingly, the cell line most susceptible to killing by both viruses was HepG2 human hepatocellular carcinoma which is reported to be mir122 negative [27]. Given that decreases in mir122 levels in primary hepatomas is an indicator of poor prognosis [28], HepG2 cells represented a promising tumour model which should maximise the therapeutic index achieved by Ad5mir122 between tumour tissue and primary liver.

### Pharmacodynamics-led determination of optimal virus doses

The optimal doses of Ad5mir122 and Ad5WT were determined in order to define a protocol that would allow repeat delivery in a cancer efficacy model. Although we have previously defined the single injection maximum tolerated dose in normal mice of Admir122 (6×10^10^ vp), treatment of xenograft bearing nude mice may require a different protocol, and therefore a pharmacodynamics-led dose escalation study was performed.

**Figure 3:**
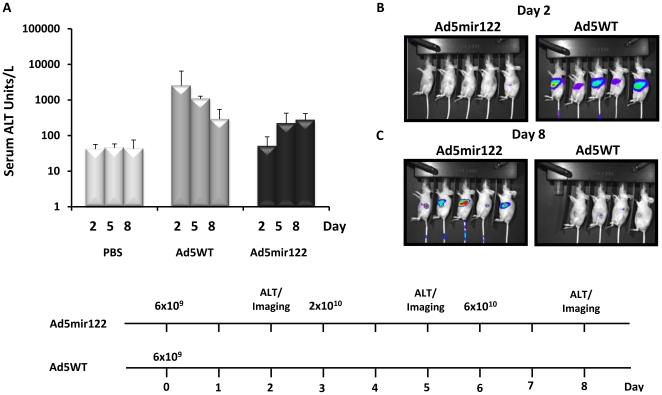
Pharmacodynamics led dose escalation study to determine the optimal treatment dose with Ad5mir122 and Ad5WT in tumour bearing mice. A) Serum Alanine Transaminase (ALT) levels from mice receiving Ad5mir122, Ad5WT or PBS. ALT values for each group are shown at days 2, 5 and 8 (n = 3) after the first injection. Data is presented as ALT units per litre using the equation in the materials and methods. B) Luciferase imaging 2 days after the first injection of Ad5mir122 (left panel) or Ad5WT (right panel). C) Luciferase imaging eight days after the first injection. Mice which received a single injection of Ad5WT are shown in the right panel and mice receiving three injections of Ad5mir122 are shown in the left panel. Images are all presented on the same scale. D) Dosage and treatment schedule for Ad5mir122 and Ad5WT. Viral doses are indicated above each line and include 10% of a Luciferase reporter virus (Ad5mir122luc). All mice were treated with bisphosphonate liposomes at day -1.

Ad5WT and Ad5mir122 were administered intravenously to CD1 nude mice bearing HepG2 xenografts, with serum ALT measured after each dose (Figure 3a). The starting dose for both viruses was 6×10^9^ vp/mouse, corresponding to 8.8×10^8^ pfu for Ad5WT, slightly lower than the published maximum tolerated dose (MTD, 1×10^9^ pfu) [17]. To enable imaging of virus activity each dose contained 10% Ad5mir122luc.

Two days following administration of 6×10^9^ vp Ad5WT, mice showed dramatically elevated ALT levels (>1000 Units/L) suggesting significant hepatic toxicity (Figure 3a). Imaging showed high levels of hepatic luciferase expression, confirming virus activity in the liver with little to no tumour signal (Figure 3b). Although the ALT values fell steadily over days 2–8 (Figure 3a), several animals (6 out of 10) showed significant weight loss (>5%) and one was put down (weight loss >15%). Accordingly this dose of Ad5WT was taken as the MTD, and treatment was not escalated.

In contrast, two days following administration of the same dose (6×10^9^ vp) of Ad5mir122, mice showed ALT readings similar to PBS controls (Figure 3a). Imaging data confirmed this result with no detectable hepatic luciferase (Figure 3b). Little to no tumour infection was observed with this dose.

The dose of Ad5mir122 was therefore increased by a half log to 2×10^10^ vp, three days after the first injection. Imaging at day five showed some luciferase activity (indicating expression of E1A) in both tumour and liver, although absolute values varied between mice (data not shown). Average ALT values showed an increase to 222 ALT units/L compared to 42 ALT units/L from controls (Figure 3a). On day six the dose was further increased by a half log to 6×10^10^ vp. Imaging at day eight showed significant luciferase activity in the tumours, however this was coupled with measurable luciferase activity also in liver (Figure 3c). ALT readings showed only a small increase from the previous dose (Figure 3a). This study confirmed that 6×10^10^ vp of Ad5mir122 was close to the MTD for Admir122, in agreement with our previous data in normal mice.

Repeat administration of Adenovirus can cause cumulative toxicity [10]. For assessment of efficacy it was desirable to administer virus on multiple occasions, hence for repeated administrations we selected dose levels that were half a log lower than the estimated MTDs of Ad5mir122 (2×10^10^ vp/mouse) and Ad5WT (2×10^9^ vp/mouse).

### Efficacy of Ad5mir122 and Ad5WT in a hepatoma xenograft model

**Figure 4:**
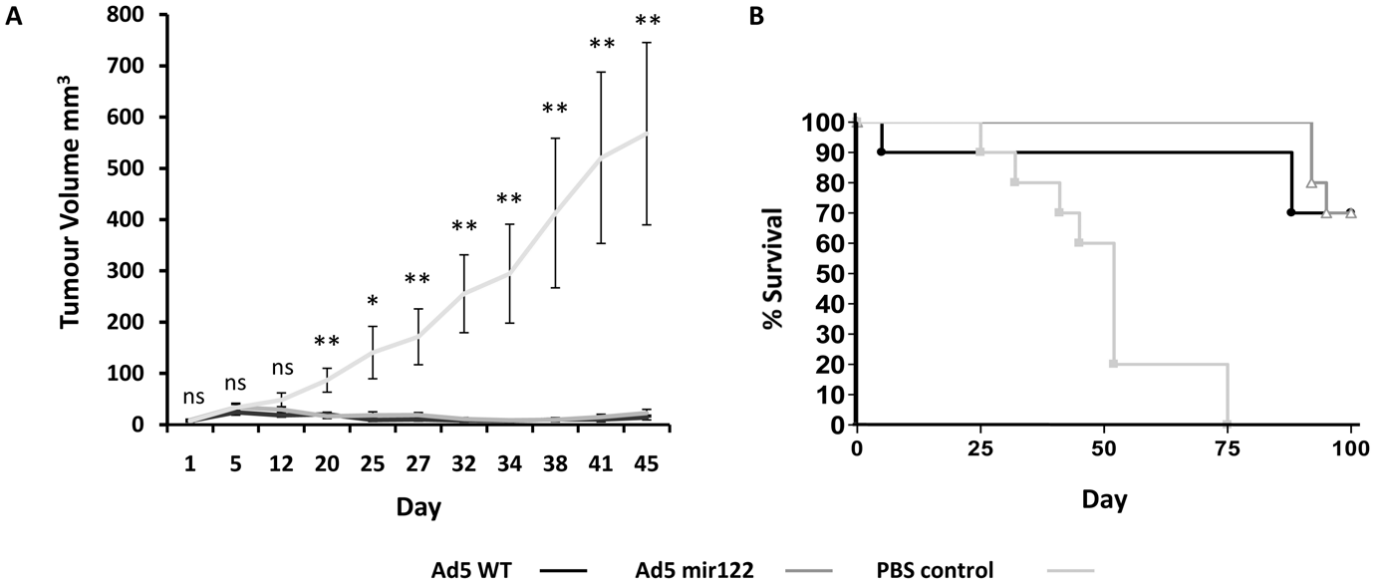
Ad5mir122 has potent anti-cancer efficacy in HepG2 tumour bearing mice. Repeat administration of Ad5mir122 (n = 10 mice) at the treatment dose determined from figure 2 (2×10^10^vp) on days 0, 3, 19 and 22 by intravenous injection. Control animals received repeat administration of 2×10^9^ vp Ad5WT or PBS by intravenous injection (n = 10 mice). A) Tumour volume of mice receiving PBS or Ad5mir122 or Ad5WT was calculated as the volume of an ellipsoid [24]. Statistical analysis was performed using one way ANOVA with a Bonferroni post test (ns  =  not significant, *  = P<0.05, **  = P<0.01) at each time point. Error bars are shown as standard error. B) Kaplan Meier survival analysis shows increased survival of mice receiving Ad5mir122 and Ad5WT compared to control mice receiving PBS. After 4 days in treatment with Ad5WT one mouse was put down due to toxicity. No treatment related toxicities were observed during Ad5mir122 treatment or treatment with PBS. Days shown on both of the horizontal axis represent the number days since the first virus injection.

To determine the anti-cancer activity of Ad5mir122, nude mice bearing established HepG2 xenografts were administered 2×10^10^ vp intravenously on days 0, 3, 19 and 22 while control animals received either 2×10^9^ vp of Ad5WT or PBS. Treatment began with tumours between 10–20 mm^3^ in size. Tumour growth was monitored for efficacy and a survival endpoint (for the Kaplan Meier graph) was introduced when individual tumour volume reached 400 mm^3^. Animals showing the fastest tumour growth were allowed to continue beyond this point (up to 1000 mm^3^) to allow the group average tumour size to reach 400 mm^3^. Mice administered Ad5mir122 showed significantly reduced tumour volume from day 20 compared to PBS controls (Figure 4a). However, mice receiving Ad5WT also demonstrated significant anti-cancer efficacy. This is perhaps unsurprising given that Ad5WT has been shown to have potent anticancer activity [29]. It is important to highlight that the doses of Ad5mir122 and Ad5WT were not equitoxic. After 4 days in treatment with Ad5WT one mouse was put down due to weight loss (>15%) and hepatic toxicity (Figure 4b). No such adverse events occurred in the group receiving Ad5mir122 at a 10-fold higher dose.

Kaplan Meier analysis of mice administered Ad5mir122 showed increased survival with all mice surviving longer than all controls (Figure 4b). These data show that the repeat administration of Ad5mir122 at doses above the MTD of Ad5WT can have significant anti-cancer efficacy without toxicity.

### Assessment of intra-hepatic Ad5mir122 E1A activity

**Figure 5:**
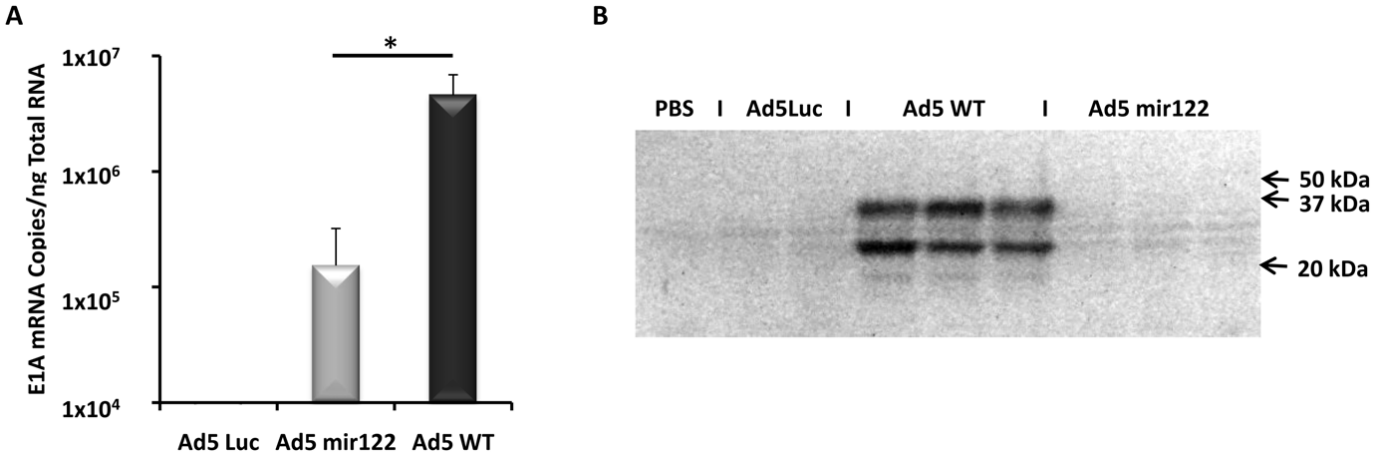
MicroRNA mediated knockdown of both E1A mRNA and protein measured by RT-QPCR and western blot after intravenous injection of 2×10^10^ vp of Ad5mir122. A) RT QPCR for the 13S E1A mRNA transcript in the livers of mice 48 hrs after intravenous injection with 2×10^10^ vp of Ad5mir122, Ad5WT, Ad5Luc or PBS. Ad5mir122 shows significantly reduced E1A mRNA during liver infection when compared to Ad5WT (N = 3). Statistical analysis was performed using a two tailed student T-Test (*P = <0.05). B) Western blot to confirm that all E1A proteins variants are knocked down. Each lane represents protein extracted from an individual mouse. Control lanes containing liver from mice treated with either an E1A deleted Ad5Luc vector or PBS show no E1A signal. Ad5WT treatment shows 3 clearly defined bands corresponding to proteins produced from the 13S (36 kDa), 12S (26 kDa) and a smaller fainter band which may represent the 11S or the 10S E1A transcript product. Treatment with Admir122 shows significant knockdown in E1A protein levels for all splice variants. The blot was exposed for 1, 5 and 10 minutes with the 5 minute exposure presented here. Molecular weights were calculated against a dual colour molecular weight ladder (Bio-Rad).

To quantify both the E1A mRNA and protein produced by Admir122 at the optimal treatment dose in comparison to Ad5WT, Balb/C mice were injected with 2×10^10^ vp and the livers were harvested after 48 hrs. As further controls mice were administered PBS or an E1A deleted non-replicating adenovirus type 5 encoding luciferase (AdLuc) at the same dose. RNA was extracted and the major E1A 13S transcript was measured by RT-QPCR. Ad5mir122 showed a 29-fold decrease in the level of 13S E1A transcript copies when compared to Ad5WT. E1A mRNA copies per nanogram of total RNA were 4.57×10^6^ and 1.54×10^5^, respectively. This confirmed that the stability of the mRNA is itself directly affected by microRNA regulation, as would be expected (Figure 5a).

To confirm that the reduced level of E1A mRNA resulted in decreased E1A protein production, and to confirm that the effect was not E1A 13S specific, liver protein extracts were analysed by western blot for E1A protein. Figure 5b shows that whilst infection with Ad5WT resulted in significant E1A protein production, infection with Ad5mir122 resulted in almost undetectable levels. The major species of E1A protein correlating with the 36 kDa (13S) and 26 kDa (12S) proteins are clearly visible with a smaller band which may represent the protein product of the 10S or 11S mRNA. No E1A signals were detected in control mice receiving PBS or a non-replicating E1A deleted AdLuc vector. Equal protein loading and blot transfer were confirmed by Ponceau staining (data not shown).

### Mir122 levels remain unaffected by Ad5mir122 infection

**Figure 6:**
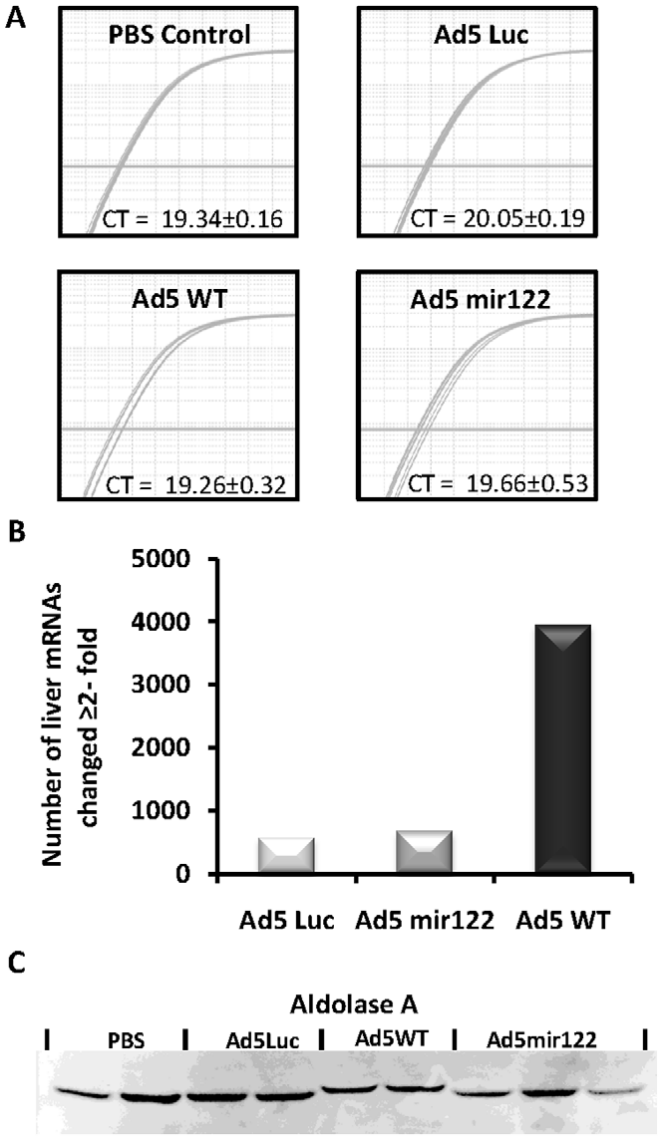
The level and activity of mature mir122 *in vivo* is unaffected by Ad5mir122. Mice were injected with 2×10^10^ vp of either Ad5mir122, Ad5WT, an E1A deleted Ad5luc vector or PBS. Quantification of mir122 mature RNA levels was performed using a Taqman microRNA assay specific for mir122. CT values were corrected against the levels of the microRNA, let7a, as a reference gene using the method published by Pfaffl M [25]. A) RT-QPCR for mir122 showing nine superimposed amplification curves (from three mice) in each treatment group, before correction against let7a. Samples were reverse transcribed using equal amounts of total RNA (5 ng) and RT-PCR was performed using equal amounts of cDNA. CT values shown here represent the average of the reactions from three mice plus or minus standard deviation. B) The total number of mRNA changes recorded by genome wide mRNA profiling of extracted murine hepatic RNA. Positive signals are those in which the median mRNA level changed by ≧2-fold from all mice in each group in comparison to median mRNA level in mice treated with PBS (n = 3 for all groups). The total number of genes altered is calculated using the average of the three independent replicates and therefore no error bars are shown. C) Western blot analysis of the mir122 regulated protein Aldolase A in mice treated as above. Liver protein extracts were subjected to a BCA protein assay and equal loading was confirmed by Ponceau stain (data not shown).

The production of E1A mRNA within hepatocytes that contains multiple microRNA binding sites could lead to the sequestration of mir122 away from its endogenous mRNA targets or depletion of the total mir122 content in the cell. The livers screened in figure 5 by E1A western blot and RT-QPCR were analysed to determine the quantity of mature mir122 RNA. All results were standardised against the highly abundant microRNA let7a which should be unaffected by Admir122 treatment. Comparative analysis of the level of mir122 found in mice treated with Ad5WT, Ad5mir122 or Ad5luc showed that there was no difference in the levels of mir122 following any virus administration when compared to the level of mir122 in mice administered PBS (Figure 6a). The ratio of mir122 levels in mice administered Ad5WT, Admir122 and Ad5luc when compared to the amount in mice receiving PBS were 1.04 (±0.188), 1.06 (±0.163) and 1.14 (±0.14), respectively. This proved that the level of mir122 was unaffected by the presence of Ad5mir122 infection however it did not confirm if mir122 was being sequestered away from its endogenous mRNA targets by direct competition.

### Ad5mir122 reduces genome wide hepatocyte mRNA changes

Microarray genome wide mRNA profiling was performed on livers of mice 48 hrs after the injection of either 2×10^10^ vp of Ad5mir122, Ad5WT, an E1A deleted non-replicating AdLuc vector or PBS. This assessment allowed the quantitation of mRNA changes due to microRNA regulation of Ad5mir122 but also served to highlight any mRNA differences between Ad5mir122 and Ad5WT. The maximum change observed in any mRNA was 111-fold although any mRNA profile change greater than 2-fold was considered significant and highlighted for further analysis. Data from the three independent animals was remarkably consistent and can be viewed at the NCBI's Gene Expression Omnibus database. The most striking result between the different viruses was the total number of mRNAs changed. Whilst Ad5WT altered 3946 mRNAs ≥ 2-fold, Ad5mir122 altered only 668 mRNAs (Figure 6b). The E1A deleted AdLuc vector altered a total of 564 mRNAs ≥2-fold. This shows that the technique can detect many changes of varying magnitude but that infection of murine liver with Ad5mir122 causes significantly fewer intracellular changes than infection with Ad5WT.

The presence of additional microRNA regulated mRNA could lead to sequestration of mir122 molecules away from their endogenous targets. Therefore, any mRNA change observed between mice administered PBS and Ad5mir122 that was not shared between Ad5mir122 and either Ad5WT or AdLuc were considered Ad5mir122 specific and were investigated further. These mRNAs, which numbered only 21 in total (Table 1), were compared with the predicted mir122 targeted mRNAs in the European Bioinformatics Institutes (EBI) Microcosm database. No transcripts were predicted to contain mir122 binding sites. The list of mir122 microcosm predicted targets was then compared to all the signals from the microarray profiling to make sure no important signals had been omitted by filtering the results as described above. 143 predicted mir122 targets were altered in the microarray analysis. Of those 143 mRNAs, 108 were altered only by Ad5WT, 20 were shared by Ad5mir122 and Ad5WT but were altered to the same extent and in the same direction (up- or down-regulated). The remaining signals showed AdLuc specific changes. This data shows that during the window of initial infection with Admir122 the presence of microRNA regulated viral mRNA does not alter the level of the endogenous microRNA mRNA targets.

**Table 1:** mRNAs in which the level was changed ≧2-fold by Ad5mir122 infection.

*Gene Symbol*	*Compared to PBS*		*Compared to Ad Luc*		*Compared to Ad5 WT*		*Gene Name*	*Accession*
LOC381748	4.9	↓	5.6	↓	2.7	↓		XM_355738.1
2310016F22Rik	4.6	↓	5.2	↓	2.6	↓	RIKEN cDNA 2310016F22 gene (2310016F22Rik)	NM_173743.2
Irf1	4.3	↓	5.0	↓	2.3	↓	Interferon regulatory factor 1 (Irf1)	NM_008390.1
Trim21	3.8	↓	4.2	↓	2.2	↓		NM_009277.2
A630097K09Rik	3.5	↓	3.7	↓	2.5	↓		AK042505
C2ta	3.4	↓	3.3	↓	2.2	↓		AK040723
LOC435565	3.4	↓	5.2	↓	2.4	↓	Similar to interferon-inducible GTPase (LOC435565)	NM_001013828.1
Trim34	3.1	↓	3.4	↓	2.2	↓	Tripartite motif protein 34 (Trim34)	NM_030684.1
Tapbpl	3.1	↓	3.3	↓	2.1	↓	TAP binding protein-like (Tapbpl)	NM_145391.1
Trim34	3.0	↓	3.3	↓	2.0	↓	Tripartite motif protein 34 (Trim34)	NM_030684.1
Iigp-pending	2.9	↓	3.4	↓	2.0	↓		AK089702
H2-T23	2.8	↓	3.5	↓	2.1	↓	Histocompatibility 2, T region locus 23 (H2-T23)	NM_010398.1
Scotin	2.6	↓	2.8	↓	2.2	↓	Scotin gene (Scotin), transcript variant 2	NM_026381.1
Saa1	2.4	↓	2.5	↓	2.0	↓		NM_009117.1
EG240327	2.4	↓	2.8	↓	2.4	↓	Predicted gene, EG240327 (EG240327)	NM_001033767.1
LOC280487	2.3	↑	2.1	↑	3.6	↑		XM_203701.3
1200013B22Rik	2.3	↓	3.0	↓	2.2	↓	NUAK family, SNF1-like kinase, 2 (Nuak2)	NM_028778.2
Tapbp	2.2	↓	3.0	↓	2.9	↓	TAP binding protein (Tapbp), transcript variant 1	NM_001025313.1
0710001B24Rik	2.1	↓	3.0	↓	2.0	↓		NM_175118
LOC244882	2.1	↓	2.2	↓	2.2	↓		XM_146997.2
Tnfaip8l3	2.0	↓	2.0	↓	2.1	↓	Tumor necrosis factor, alpha-induced protein 8-like 3 (Tnfaip8l3)	NM_001033535.1

Total mRNA profiling produced 4562 signals in which the mRNA levels were altered ≧2-fold after administration of either 2×10^10^ vp of Ad5WT, Ad5mir122, an Ad5luc control vector or injected with PBS. The mRNAs in this table are those that show at least a 2-fold change in quantity following Ad5mir122 infection that are not altered when compared to mice administered PBS, Ad5luc or Ad5WT. Mir122 predicted target mRNAs (produced using the Microcosm database) were compared to this list but no positive matches were found.

### Mir122 regulated Adolase A levels remain unaffected by Ad5mir122

Although no differences had been observed in the levels of known mir122 regulated mRNAs it was unclear if the quantity of protein produced from these transcripts was altered. MicroRNA suppression has been shown to decrease the protein level produced from some mRNAs without changing the mRNA steady state levels [30]. Therefore, mice were injected with 2×10^10^ vp of Ad5mir122, Ad5WT, AdLuc or PBS. After 72 hours livers were analysed by western blot for the levels of Aldolase A protein, a known mir122 target [31], [32]. Inhibition of mir122 function is known to increase Aldolase A protein levels although the western blot showed no difference between mice in the different treatment groups (Figure 6c). This data suggests that mir122 regulation of Aldolase A is maintained despite the presence of Ad5mir122 at therapeutic doses.

## Discussion

MicroRNA regulation of viral gene expression and replication exploits the negative regulatory principle of microRNA mRNA degradation. The success of this approach is reflected in the number of RNA viruses for which it has already proven successful [5], [7], [8], [33], [34]. In creating a DNA virus which was microRNA regulated we anticipated that the level of control would be less efficient and potentially more toxic by interfering with the microRNA regulation of endogenous mRNA targets. This was expected because the viral genome itself is not directly destroyed when using a DNA virus and therefore continually produces microRNA targeted viral mRNA. This could lead to sequestration of the microRNA away from its endogenous mRNA targets and alter the expression patterns of many genes.

In this study we have shown that the inclusion of four perfectly complementary binding sites for hepatic specific microRNA mir122 into the 3′ UTR of adenovirus E1A allows efficient mir122-controlled expression of E1A in murine liver, confirmed by RT-QPCR and western blotting. We have demonstrated that both the quantity of the microRNA and the stability of microRNA targeted mRNAs are unaffected by Ad5mir122. Both of these results are important toxicological findings and increase our understanding of the safety profile of microRNA regulation.

Interestingly, the genome wide profiling of the mRNA levels in livers infected with Ad5mir122 produced 21 mRNAs that were altered ≧2-fold but it was not clear that they were directly regulated by mir122. These signals may represent experimental noise, however, given the reproducibility of the signals in comparison to all other treatment groups and the extent to which they are altered (in some cases up to 5-fold) this seems unlikely. Further investigation would be required in order to determine the cause of these changes and the effects they may have on cell function.

The data reported here suggests that the highly abundant microRNA mir122 is capable of regulating more mRNA transcripts than are regulated under normal conditions in hepatocytes and that it is probably expressed in excess of its regulatory requirements. This was an advantage in our studies as it allowed tight regulation of adenoviral E1A without altering endogenous mRNA levels. However, other microRNAs may not be as successful at controlling additional mRNA, especially if they are expressed at lower quantities. The direct competition for microRNA-mRNA binding between endogenous and exogenous viral mRNA could result in changes in protein expression if the quantity of the microRNA required for normal regulation was only just met by its expression level. It has been shown that significant alterations to the microRNA machinery can have toxic side effects, usually by interfering with total microRNA production [35] (i.e. DICER or RISC processing). Given that microRNAs do not appear to have any obvious feedback mechanism to report the addition of new target mRNA molecules, it seems unlikely that increasing the quantity of mRNA target, as was done in this study, for a single microRNA will cause such broad toxicity.

Within this paper we have also shown that the intravenous administration of repeat doses of Ad5mir122 mediates anticancer activity with negligible toxicity. All animals receiving Ad5mir122 performed better than all controls. This is the first anti-cancer efficacy reported using a microRNA regulated oncolytic DNA virus. Our data also builds on previously published evidence that Ad5WT has potent anticancer efficacy [29], even at low doses. However, as was clear in our studies, toxicity is a major limitation to its use in animal models.

Genetic modification of adenoviruses to allow replication selectively within tumour cells often relies on their activation by cellular mechanisms, which can be weak compared to intrinsic viral mechanisms. Accordingly such viruses, while providing good safety, often show decreased anti-cancer potency compared to wild type strains, particularly *in vivo* [29]. The exploitation of the microRNA regulatory network is enabling the design of many new viruses for cancer virotherapy, vaccination and gene therapy that are modified to allow selective replication and expression only in specific cell types and represents a new, safe and effective method of controlling of viral gene expression.
